# Influence of the Layer Directions on the Properties of 316L Stainless Steel Parts Fabricated through Fused Deposition of Metals

**DOI:** 10.3390/ma13112493

**Published:** 2020-05-29

**Authors:** Takashi Kurose, Yoshifumi Abe, Marcelo V. A. Santos, Yota Kanaya, Akira Ishigami, Shigeo Tanaka, Hiroshi Ito

**Affiliations:** 1 Research Center for GREEN Materials & Advanced Processing, Yamagata University, 4-3-16 Jonan, Yonezawa, Yamagata 992-8510, Japan; takashi.kurose@yz.yamagata-u.ac.jp; 2 Department of Organic Materials Science, Graduate School of Organic Materials Science, Yamagata University, 4-3-16 Jonan, Yonezawa, Yamagata 992-8510, Japan; tcs89036@st.yamagata-u.ac.jp (Y.A.); vergne_marcelo@taisei-kogyo-net.co.jp (M.V.A.S.); akira.ishigami@yz.yamagata-u.ac.jp (A.I.); 3 Taisei Kogyo Co. Ltd., 26-1 Ikeda-Kitamachi, Neyagawa, Osaka 572-0073, Japan; yota_kanaya@taisei-kogyo-net.co.jp (Y.K.); shigeo_tanaka@taisei-kogyo-net.co.jp (S.T.)

**Keywords:** fused deposition of metals, 316L stainless steel, layer directions

## Abstract

Metal specimens were fabricated via the fused deposition of metals (FDMet) technique with a filament composed of the 316L stainless steel particles and an organic binder. This process was adopted due to its potential as a low-cost additive manufacturing process. The objective of this study is to investigate the influence of the processing conditions—layer directions and layer thicknesses—on the mechanical and shrinkage properties of the metal components. The specimens were printed in three different layer directions. The highest ultimate strength of 453 MPa and strain at break of 48% were obtained in the specimen printed with the layer direction perpendicular to the tensile direction. On the other hand, the specimen printed in the layer direction parallel to the tensile direction exhibited poor mechanical properties. The reason for the anisotropy of the properties was investigated through systematic SEM observations. The observations revealed the presence of segregated binder domains in the filaments. It was deduced that the binder domain was oriented in the direction perpendicular to that of the layer and remained as oriented voids even after sintering. The voids oriented perpendicular to the tensile direction act as defects that could cause stress concentration, thus resulting in poor mechanical properties.

## 1. Introduction

Additive manufacturing (AM) has been extensively employed in the fabrication of complex-shaped parts that are difficult to achieve through the traditional machining processes. It has also been utilized in making products without mold and material losses [1,2]. Metal AM techniques such as electron beam melting (EBM) [3,4,5,6] and direct metal deposition [7,8] have been studied and developed for academic and industrial applications such as biomedical field, aerospace, and military. However, these metal AM techniques operate in inert gas environments and require cooling systems, hence they have high installation and maintenance costs. They also demand high energy to melt metallic powders. Therefore, alternative low-cost metal AM techniques are desired.

Various AM techniques that have the potential to be low-cost processes have been proposed. Such techniques include the fused deposition of metals (FDMet) [9,10] and composite extrusion modeling (CEM) [11,12]. The FDMet process is divided into four steps. The first involves the preparation of the raw material, which consists of metal powder particles and thermoplastic organic binder. The prepared material is termed the “feedstock.” The second step involves the fabrication of the filaments by extruding the feedstock. In the third step, the 3D part is printed; the part is termed “green part.” The fourth step involves the removal of the organic binder from the green part via debinding processes; the obtained part is termed “brown part.” Thereafter, the brown part is sintered to obtain the part termed the “silver part.” The processes above combine mainly two different technologies. One is the fused deposition modeling (FDM) process [13,14] where a predefined digital 3D object is built layer by layer with an extruded thermoplastic filament by a moving extruder. The FDM is a widely used process, and it can significantly reduce the production cost. The other technology is the metal injection molding (MIM), a powder metallurgical process [15,16], where metal particles are sintered together at a temperature well below their melting points, thus, less heat energy is consumed.

Currently, the FDMet-type printing systems are manufactured by a few companies (e.g., Desktop Metal, Inc. and Markforged, Inc.). The filaments for the conventional FDM 3D printers are also available (BASF 3D Printing Solutions GmbH, Ultrafuse 316L). Thus, new technology is becoming widely available. For functional or structural applications of the produced metallic components, the relationship among the material printing process conditions, internal structure, and properties—mechanical properties and dimensional stability—need to be deeply understood. Considering that the green parts are fabricated with many interfaces in the FDM process, special attention should be paid to the relationship between the interfaces and the mechanical properties. However, the commercially available FDMet systems mentioned above are “closed-access,” i.e., they do not allow users to set arbitrary fabrications like setting the process conditions and building a specimen without a predefined pedestal. Although several studies on the FDMet process have already been published [12,17,18,19], none of them have reported the relationship between the mechanical properties of the printed parts and the layer directions.

The objective of this study is to investigate the influence of the processing conditions—the layer directions and layer thickness—on the mechanical properties and shrinkage of printed products. The conventional feedstock used in MIM was employed in this study. The 316L stainless steel particles were used, due to their excellent corrosion resistance, high strength, and biocompatibility. An organic binder system that can be removed by a thermal debinding process was employed. The specimens were printed in three different layer directions. The study revealed that the layer directions have a significant influence on the mechanical properties of the printed part. Only the specimen whose layer direction was parallel to the tensile test direction exhibited poor mechanical properties. The mechanism behind the effect was investigated by systematic and detailed internal structural observations.

## 2. Materials and Methods

The feedstock, composed of 60 vol% of 316L stainless steel particles (average particle diameter of 10 µm) and 40 vol% of organic binder composed of polyoxymethylene and paraffin wax, was obtained from Taisei Kogyo Co. Ltd., (Osaka, Japan). The melt temperatures of the paraffin wax and polyoxymethylene were 62 and 165 °C, respectively. The decomposition completion temperature of the organic binder was 460 °C. The filament was prepared in a capillary rheometer with a barrel of diameter 9.5 mm and a die of diameter 1.75 mm (CAPILOGRAPH-1D, Toyo Seiki Seisaku-syo, Ltd., Tokyo, Japan) by extruding the feedstock vertically with a piston at a temperature of 130 °C and extrusion rate of 50 mm/min. The extruded filament was wound up below the die and cooled to room temperature. A filament of diameter 1.73 mm ± 0.02 was obtained.

**Figure 1:**
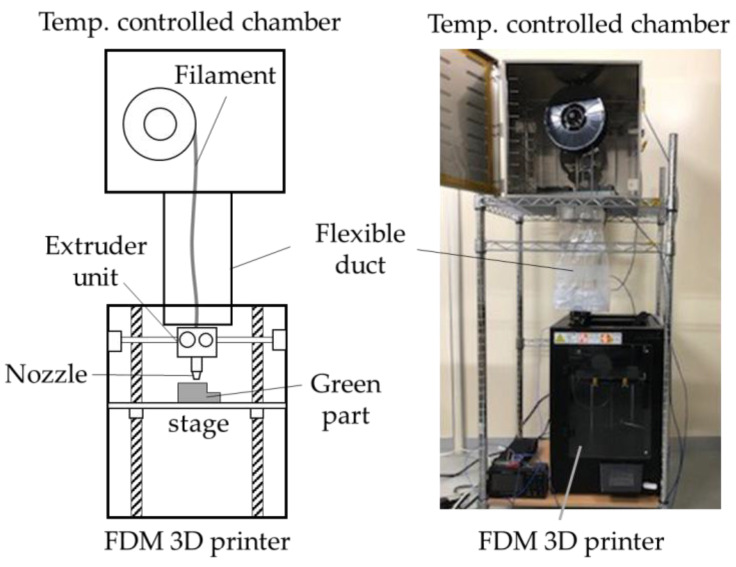
Overview and a picture of the 3D-printing system.

**Figure 2:**
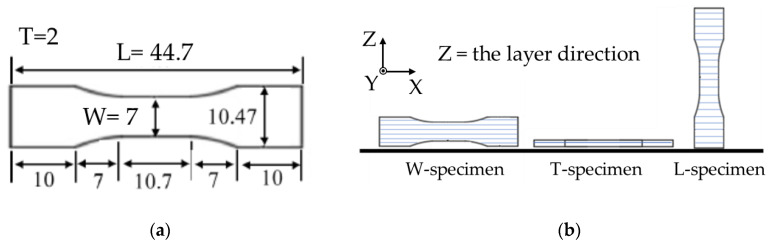
(**a**) Dimension of a specimen. (**b**) Layer directions of various specimens and the coordinate axes.

The 3D-printing system is shown in Figure 1. A conventional FDM 3D printer for plastic filaments (L-DEVO M2030TP, Fusion Technology Co., Tokyo, Japan) was modified to be employed in this study. Because the filament was brittle and was not to be supplied to the printer at a temperature less than 70 °C, a temperature-controlled chamber was connected to an extruder unit through a flexible duct to control the temperature of the filament. The printing conditions are listed in Table 1. Two conditions of layer thicknesses at 0.1 and 0.3 mm were examined. The dimensions of the designed specimens are shown in Figure 2a. The specimens were printed in three different layer directions; Figure 2b shows the definition of specimens together with the coordinate axes. The extruder unit with the nozzle moves only in the X-Y plane, and the stage moves only in the Z direction. The layer direction is in the *Z*-axis direction of a specimen. All layers were printed nominally at 100% infill density with a rectilinear infill pattern [20]. To print each layer, two outside lines were first printed, and then, the inside was filled at a raster angle of ± 45° alternating in each layer (i.e., layer n − 1/n/n + 1 = 45°/−45°/45°) [20]. The 3D printed parts were termed the “green parts.”

Thermal debinding and sintering processes were carried out in the same vacuum furnace (Shimazu industrial systems Co. Ltd., Otsu, Shiga, Japan). Firstly, the green parts were subjected to thermal debinding at 600 °C for 2 h under a nitrogen atmosphere to remove the organic binder. Thereafter, they were sintered at 1280 °C for 2 h in an argon atmosphere. Irrespective of the layer directions, all specimens were positioned on a flat carbon plate in the furnace such that the thickness was parallel to the direction of gravity. The sintered parts were termed the “silver parts.”

The densities of the silver parts were estimated from the weights in water and air using the Archimedes principle. Their relative densities were calculated thereafter. The theoretical density of 7.98 g/cm^3^ for 316L stainless steel was used for the calculations [21]. The dimensions of green and silver parts were measured (using a caliper) from which the linear shrinkages from the green part to the silver part were estimated. The tensile test was performed using a universal testing machine with a load cell of 5kN (Autograph AGS-X, Shimazu Corp., Kyoto, Japan). The crosshead speed was 2 mm/min. Three samples of each specimen were tested to check the repeatability.

The internal structures of the feedstocks, filaments, green parts, and silver parts were analyzed. The fracture surface of the feedstock and filament were prepared at room temperature and observed under an SEM (TM3030 plus, Hitachi High-Technologies Corp., Tokyo, Japan). The polished cross-sections of the green parts and the silver parts were also observed under the SEM. The specimens were embedded in epoxy resin, after which they were polished using a polishing machine (ML-150P, Maruto Instrument, Co. Ltd., Tokyo, Japan). For both the green and silver parts, a section perpendicular to the length of the specimen were observed. In addition, the section perpendicular to the thickness was also observed. The fractured surfaces of the silver parts after the tensile test were also observed.

## 3. Results and Discussion

### 3.1. Relative Density

**Figure 3:**
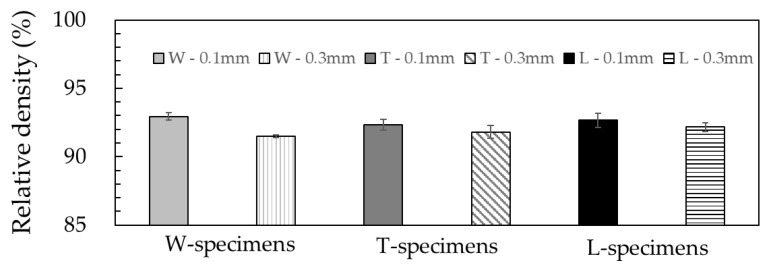
Results of relative density measurement of green parts printed in various layer directions and thicknesses.

Figure 3 shows the relative densities of the silver parts printed in various layer directions and thicknesses. Among the specimens printed in different layer directions, the parts with a layer thickness of 0.1 mm showed higher relative densities than those with a thickness of 0.3 mm. Regardless of the layer directions, similar relative densities were obtained among the specimens printed in the same thickness. The highest relative density obtained was 92.9% (at a layer thickness 0.1 mm and in W-specimen). This value is consistent with the 92.2% reported for a 316L part produced with a metal/polymer composite filament composed of 88 wt% (~55 vol%) 316L particles (particle size of 30–50 µm) in a polymer matrix (POM) [17]. The catalyst and thermal debinding processes were employed to remove the POM binder, and the part was sintered at a temperature of 1360 °C for 2 h under argon environment.

It has been known that the relative density varies with process conditions and loadings of metal particles. Thompson et al. [19] investigated the effect of debinding and sintering conditions on the relative density of 316L parts. They used a filament of 55 vol% 316L particles of diameter 17.7 µm and an organic binder. The solvent and thermal debinding processes were employed, and parts were sintered at 1330–1360 °C for 30–120 min. The relative density of the silver part was improved from 81% to 95%; the specimen sintered at 1360 °C for 2 h showed a 95% relative density. In the case of the feedstocks for MIM, it has been established that a higher amount of metal particles (more than 60 vol%) yields high relative densities of above 95% [15,22]. Based on these, we presume that the relative density of the specimens in this study could be improved by optimizing the debinding and sintering conditions and metal particle loadings.

### 3.2. Dimensional Linear Shrinkage

**Figure 4:**
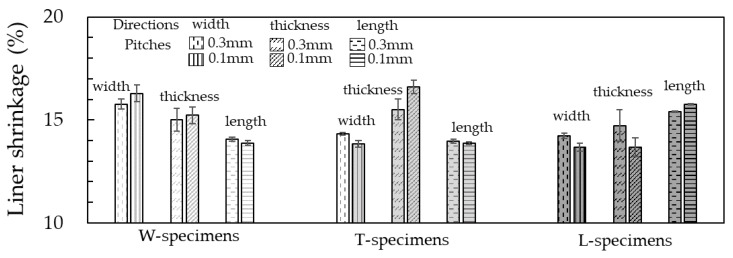
Results of dimensional linear shrinkage from green parts to silver parts printed in various layer directions and layer thicknesses.

The dimensional linear shrinkages from the green parts to the silver parts for the specimens printed in different layer directions and thicknesses are shown in Figure 4. All the specimens printed in various directions exhibited anisotropic shrinkage behavior; the linear shrinkage was highest along the layer direction. Gong et al. reported an anisotropic shrinkage behavior, from the green part to the silver part, for a specimen printed with a filament (Ultrafuse 316LX, BASF, Ludwigshafen, Germany), composed of polymer binder and 316L [18]. The parts were sintered in such a position that the layer directions were parallel to the direction of gravity. The linear shrinkages in eleven features of the part were measured; the shrinkage in the layer direction and perpendicular direction were 15–23% and 14–18%, respectively, which are similar to our results (15–17% and 14–15%, respectively). The highest shrinkage was obtained along the layer direction in all features. They attributed the anisotropic shrinkage behavior to the effect of gravity on the metal part during the sintering process. In this study, the specimens printed in the various layer directions were sintered in the same position such that the thickness of the specimen was parallel to the direction of gravity. Nevertheless, the highest shrinkages in the width and length directions were obtained in the W-specimen and T-specimen, respectively. Therefore, the anisotropic shrinkage behavior could be caused by the layered structure. The mechanism is discussed in detail in Section 3.5.

### 3.3. Tensile Test

**Figure 5:**
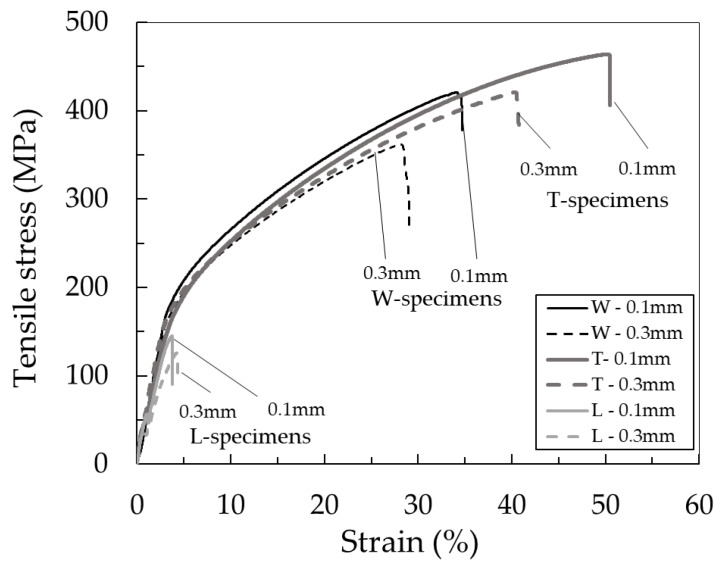
Tensile stress–strain curves of the silver parts printed in various layer directions and thicknesses.

**Figure 6:**
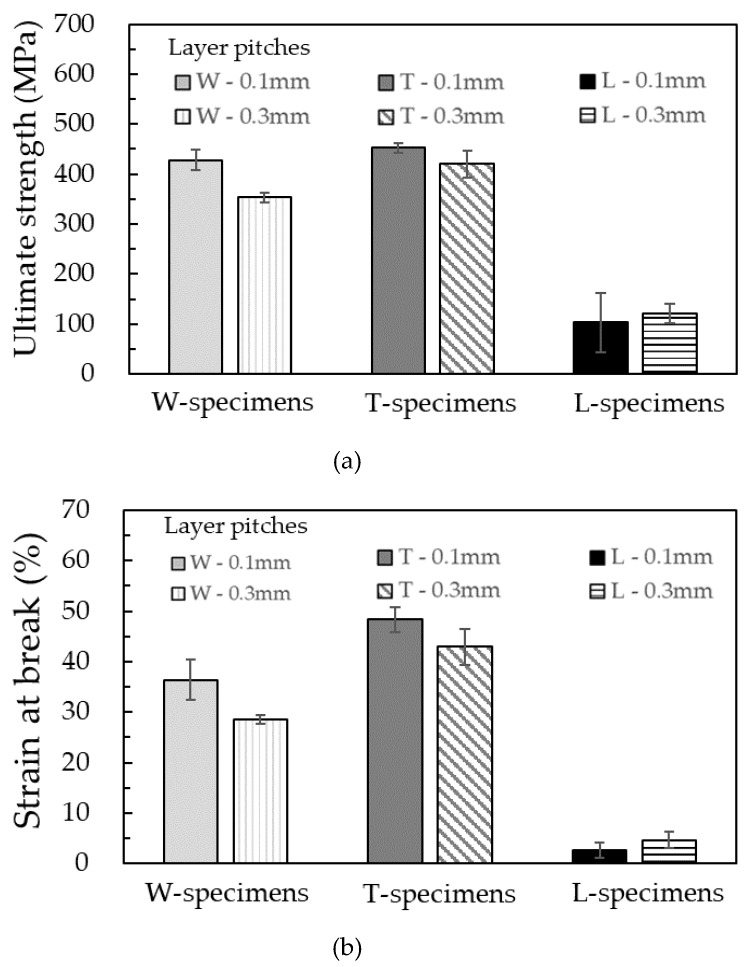
Results of tensile tests. (**a**) Ultimate strength and (**b**) strain at break of the silver parts printed in various layer directions and thicknesses.

Figure 5 shows the tensile stress–strain curve of the silver parts printed in different layer directions and thicknesses. The average ultimate strengths and strains at break of all specimens are summarized in Figure 6. The T-specimen exhibited the highest ultimate strength and strain at break, followed by the W-specimen. Among the T- and W-specimens, the specimens printed with a layer thickness of 0.1 mm showed higher ultimate strength and strain at break than those with a thickness of 0.3 mm. The highest ultimate strength of 453 MPa and strain at break of 48% were demonstrated by the T-specimen printed with a layer thickness of 0.1 mm. These values meet the standard values (tensile strength > 450 MPa, strain at break > 40%) for sintered-metal injection-molded materials issued by the Japan Powder Metallurgy Association (JPMA S01:2014) [23]. They are also consistent with those reported as 465 MPa and 31%, respectively, reported by Gong et al. [18]. In their study, the layer direction was perpendicular to the tensile direction, and the relation corresponds to T-specimen in this study. Their parts were printed with filament (Ultrafuse 316LX, BASF) composed of 316L and polymer binder with a layer thickness of 0.2 mm.

**Figure 7:**
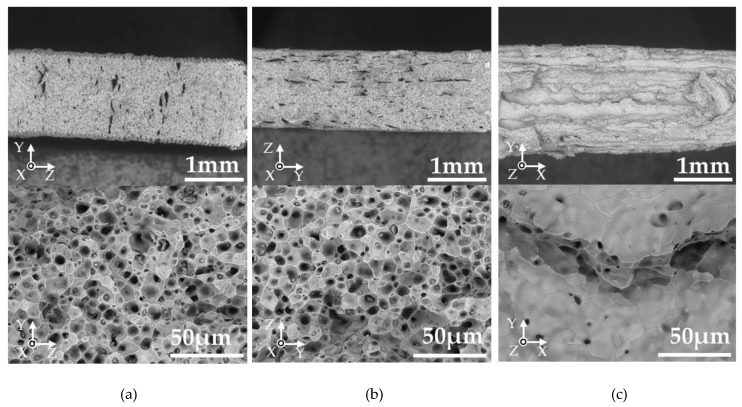
SEM micrographs of fracture surfaces after tensile tests of the silver parts printed in a layer thicknesses in 0.1 mm. Specimens are (**a**) W-specimen, (**b**) T-specimen, and (**c**) L-specimen (the micrographs below are observed in higher magnification).

On the other hand, the L-specimen showed poor ultimate strength and strain at break. To investigate the reason for decreased properties, the fracture surfaces of the samples after tensile tests were observed under the SEM (Figure 7). Typical ductile fracture surfaces with dimple patterns were obtained in both W- and T-specimens. The L-specimen clearly showed a fracture surface different from that of the W- and T-specimens; a smoother surface structure was observed in most of the fracture surfaces. To investigate the reason for the various fracture surfaces, the internal structure of the specimens at each processing step was studied.

### 3.4. The Relation between the Internal Structure and Properties

**Figure 8:**
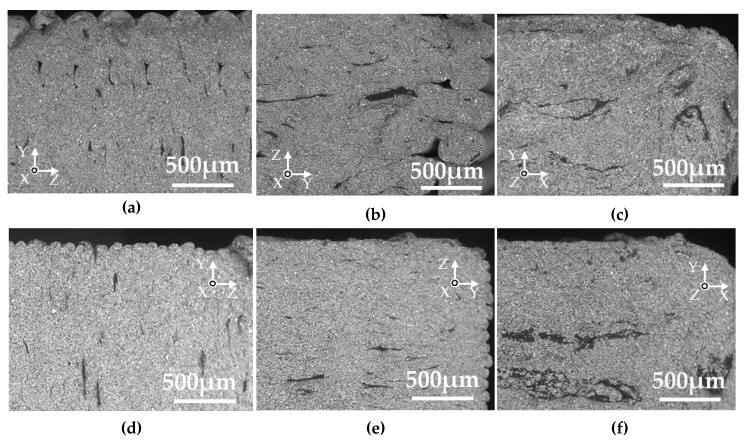
SEM micrographs of cross-sections perpendicular to the length direction of the green parts. (**a**) W-specimen, thickness 0.3 mm; (**b**) T-specimen, thickness 0.3 mm; (**c**) L-specimen, thickness 0.3 mm; (**d**) W-specimen, thickness 0.1 mm; (**e**) T-specimen, thickness 0.1 mm; (**f**) L-specimen, thickness 0.1 mm.

The cross-sections perpendicular to the length of the green parts of the various samples are shown in Figure 8. Voids were observed around the two outside lines in the W- and T-specimens with a layer thickness of 0.3 mm, but not in those with a layer thickness of 0.1 mm. Oriented domain structures were observed in the inside structure of all the specimens, which were deduced to be the binder components rather than voids based on the SEM analysis with the energy-dispersive X-ray spectroscopy (EDX). The domains were oriented perpendicular to the layer directions.

**Figure 9:**
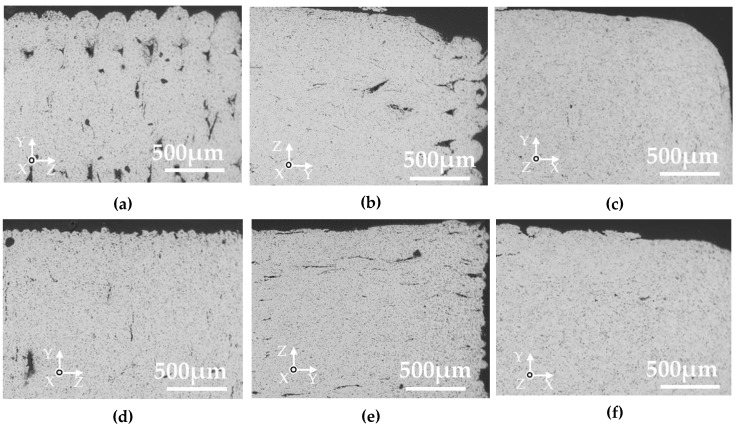
SEM micrographs of cross-sections perpendicular to the length of the silver parts. (**a**) W-specimen, thickness 0.3 mm; (**b**) T-specimen, thickness 0.3 mm; (**c**) L-specimen, thickness 0.3 mm; (**d**) W-specimen, thickness 0.1 mm; (**e**) T-specimen, thickness 0.1 mm; (**f**) L-specimen, thickness 0.1 mm.

**Figure 10:**
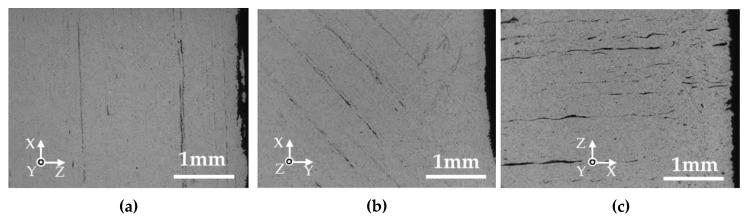
SEM micrographs of cross-sections perpendicular to the thickness of the silver parts printed in a layer thickness of 0.1 mm. (**a**) W-specimen; (**b**) T-specimen; (**c**) L-specimen.

Figure 9 shows the cross-sections perpendicular to the length of the silver parts printed in various layer directions. Voids were observed in the two outside lines and the inside of the W- and T-specimens with a layer thickness of 0.3 mm. The visible voids in the specimens with a layer thickness in 0.1 mm were smaller in size and were oriented perpendicular to the layer directions. Figure 10 shows the cross-section perpendicular to the thickness of the silver parts printed in various layer directions and a layer thickness of 0.1 mm. Many voids oriented perpendicular to the layer direction are displayed in the W- and L-specimens. Considering a tensile test where specimens were strained to the length direction, only the voids in the L-specimen are oriented perpendicular to the tensile direction. Such voids would act as fatal defects in the tensile test, thus leading to crack and fracture.

**Figure 11:**
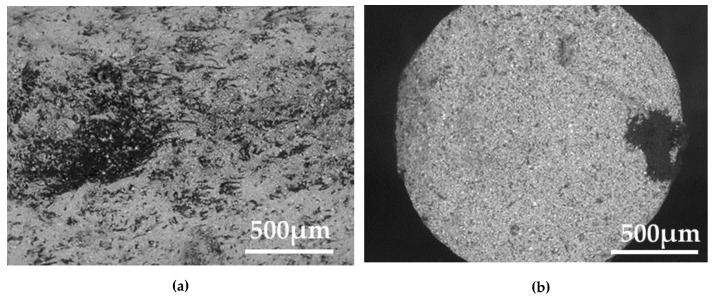
SEM micrographs of fracture surfaces of (**a**) a feedstock and (**b**) a filament.

The fracture surfaces of the feedstock and filament are shown in Figure 11a,b, respectively. The figure shows that many dark spots, less than 100 µm in diameter, were dispersed in the samples. The area with a high concentration of the spots was observed in the feedstock. The dark segregated area was also obtained in the filaments (Figure 11b). The filament was produced by extruding the feedstock with a piston at 130 °C. The processing temperature of 130 °C is less than the melting point of polyoxymethylene. Thus, the extrusion would not generate a strong mixing force, as in the screw-type extruder. Therefore, we infer that the dark segregates are aggregates of the polyoxymethylene binder.

### 3.5. The Mechanism for Anisotropic Shrinkage and Mechanical Properties

**Figure 12:**
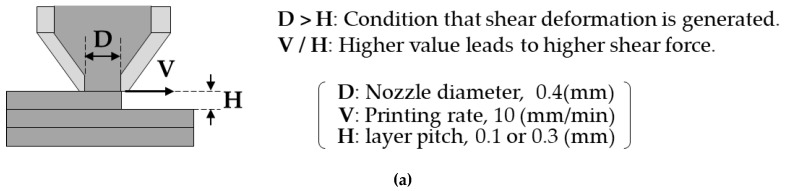

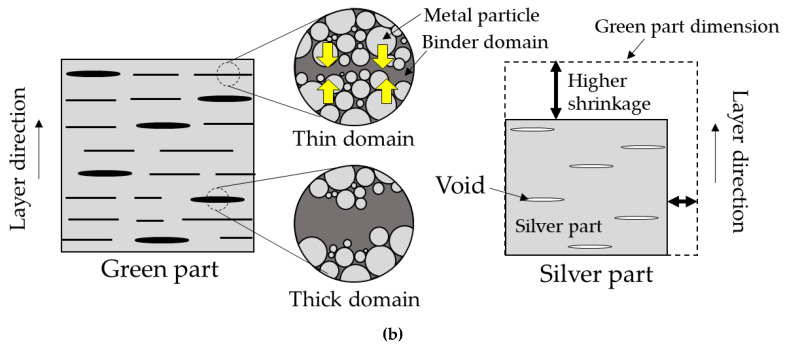
Illustration to explain the mechanism for the anisotropic shrinkage and mechanical properties. (**a**) Conditions under which shear flow is generated during the 3D print. (**b**) The effects of the oriented binder domain on the shrinkage and the generation of voids.

Based on the above, we deduced the mechanisms by which the aggregates of the binder orientate perpendicular to the layer directions, and those for the anisotropy in the shrinkage and mechanical properties. These are illustrated in Figure 12. The FDM process was performed with a nozzle of diameter 400 µm and with a layer thickness of 100–300 µm (Figure 12a). In this condition, a shear force would be applied to the extruded material by the relative displacement between the solidified underlayer and the edge of the moving nozzle. The extrusion temperature was 170 °C, which is higher than the melting temperature of polyoxymethylene, thus, the melted domain would be oriented in the direction of the moving nozzle. Furthermore, the smaller layer thickness of 0.1 mm generates higher shear strain to the material, resulting in a higher aspect ratio of the oriented region than that obtained with the layer thickness of 0.3 mm, as observed in the green parts (Figure 8).

The orientation of the binder domain perpendicular to the layer direction increased the average metal particle distance in the layer direction, which would lead to the higher linear shrinkage in the layer direction among all directions during sintering (Figure 12b). In the case where the metal particle distance in the layer direction is too long, the sintering phenomenon does not occur, hence, the binder domain area would remain as voids, as shown in Figure 9 and Figure 10. The voids oriented perpendicular to the tensile direction in the L-specimen would act as defects which could cause stress concentrations. These, therefore, give the reason for the decreased ultimate strength and the strain at break obtained in the L-specimen.

### 3.6. Features and Possibility of Metal FDMet Process

The metal part with mechanical properties that meet the industrial standards for sintered-metal injection-molded material was fabricated using the FDMet technique. This process has the potential to be a low-cost AM process because the FDM printer is low-cost, and a sintering process could require less heat energy compared with other metal AM processed such as EBM and direct metal deposition. Another advantage is that a material in the shape of a filament is easier to handle than metal powder. On the contrary, there would be a limit to the thickness and size of parts that can be manufactured using this process. Relatively thin and small-sized parts would be suitable for the technique, among other metal AM techniques because the organic binder has to be removed as a gas from a part during the thermal debinding process. The anisotropic shrinkage and mechanical properties should be thoroughly evaluated at the research and development stage; the anisotropy has to be considered during the design and manufacturing of products. Based on our results, as the existence of the binder domain was deduced to be the cause of the strong anisotropy, it should be possible to reduce the anisotropy by eliminating or reducing the binder domain through improvements in material and process technologies.

## 4. Conclusions

Metal specimens were fabricated via the FDMet technique with a filament composed of the 316L stainless steel particles and an organic binder. The influence of the processing conditions—layer directions and layer thicknesses—on the mechanical and shrinkage properties of the metal components was investigated.

The parts printed in a layer thickness of 0.1 mm showed higher relative densities than those with a thickness of 0.3 mm. The layer directions had little effect on the relative densities of the silver parts; the highest relative density obtained was 92.9%. The anisotropic shrinkage behavior was observed. The linear shrinkage was highest along the layer direction among all the specimens printed in various directions. The range of linear shrinkages in the layer direction and the perpendicular directions were 15–17% and 14–15%, respectively. The anisotropy of the mechanical property was also analyzed. The highest ultimate strength of 453 MPa and strain at break of 48% were obtained in the specimen printed with the layer direction perpendicular to the tensile direction. On the contrary, the specimen printed in the layer direction parallel to the tensile direction exhibited poor mechanical properties. The reasons for the anisotropy of the shrinkage and mechanical properties were investigated. The observations revealed the presence of segregated binder domains in the filaments. The oriented binder domain in the direction perpendicular to that of the layer was also observed in the green parts. It was deduced that the orientation of the binder domain perpendicular to the layer direction increased the average metal particle distance in the layer direction, which would cause the higher linear shrinkage in the layer direction among all directions during sintering. In the case where the metal particle distance in the layer direction is too long, the binder domain area would remain as voids even after the sintering process. The voids oriented perpendicular to the tensile direction act sensitively as defects that could cause stress concentration, thus resulting in poor mechanical properties. As the existence of the binder domain was deduced to be the cause of the strong anisotropy, it should be possible to reduce the anisotropy by eliminating or reducing the binder domain through improvements in material and process technologies.

The metal part with mechanical properties that meet the industrial standards for sintered-metal injection-molded material was fabricated using the FDMet technique. This process has the potential to be a low-cost AM process for relatively thin and small-sized parts. However, the anisotropic shrinkage and mechanical properties should be improved and considered in research and development for commercialization.

## Figures and Tables

**Table 1:** FDM 3D print conditions.

Printing Parameter	Value	Unit	Printing Parameter	Value	Unit
Nozzle diameter	0.4	mm	Printing rate	10	mm/min
Nozzle temperature	170	°C	Layer pitch	0.1, 0.3	mm
Chamber temperatuer	80	°C	Infill ratio	100	%
Stage temperature	70	°C	Outside shell thickness	0.8	mm
